# Post-partum maternal bradycardia: A case series and literature review

**DOI:** 10.1177/1753495X231178407

**Published:** 2023-07-20

**Authors:** Karen C Tran, Cassandra D Fayowski, Tessa Chaworth-Musters, Susan E Purkiss, Anthony Chau, Matthew T Bennett, Wee Shian Chan

**Affiliations:** 1 Division of General Internal Medicine, Department of Medicine, University of British Columbia, Vancouver, British Columbia, Canada; 2 Department of Medicine, Western University, London, Ontario, Canada; 3 Department of Anesthesiology, Pharmacology and Therapeutics, University of British Columbia, Vancouver, British Columbia, Canada; 4 Division of Cardiology, Department of Medicine, University of British Columbia, Vancouver, British Columbia, Canada

**Keywords:** Arrhythmia, heart rate, heart rhythm, pregnancy, pregnant, preeclampsia, hypertension, gestational hypertension, anesthesia, anesthetics, ergotamine, women

## Abstract

**Background:**

Unlike tachyarrhythmias, which are common in pregnancy, there is a paucity of data regarding maternal bradycardias. Our objective was to describe the characteristics, associated conditions, and prognosis of women who develop bradycardia post-partum.

**Method:**

We conducted a retrospective chart review of patients referred to the Obstetrical Medicine service at British Columbia Women's Hospital from January 2012 to May 2020 for post-partum maternal bradycardia.

**Results:**

Twenty-four patients with post-partum bradycardia were included (age 34.2  ±  4.8 years; heart rate 40.4  ±  8.1 beats per minute; blood pressure 131/72 mm Hg). Sinus bradycardia (79.2%) was the most common rhythm. Dyspnea (29.4%) and chest pain (23.5%) were common symptoms. Mean time to resolution of bradycardia was 3.6  ±  3.8 days. Associated conditions potentially explaining the bradycardia were preeclampsia (54.1%), underlying (16.7%), medications (8.3%), and neuraxial anesthesia (8.3%).

**Conclusions:**

Maternal bradycardia is an uncommon condition complicating the post-partum period, that is generally self-limiting, with the majority only require clinical observation.

## Introduction

Pregnancy is associated with significant physiological changes in the cardiovascular system. In response to increased metabolic needs and systemic vasodilation, maternal cardiac output can increase up to 50% during gestation.^1^ The initial increase in cardiac output results from an increase in heart rate; there is a 15–25% increase above baseline by the end of the first trimester which remains stable throughout the rest of pregnancy. The sustained increase in cardiac output in the second trimester is largely due to an increase in stroke volume.^2^

As a result of changes in circulating plasma volume and hyperdynamic circulation, pregnancy has been identified as a risk factor for supraventricular tachycardia (SVT).^3^ The most common tachyarrhythmia seen is paroxysmal SVT, and is known to occur in structurally normal hearts.^4^ By contrast, bradyarrhythmias are uncommon, with a prevalence of 1 in 20,000 for women of reproductive age.^5^ Moreover, in a large cohort of post-partum women, bradycardia is rare, with median (3rd–97th centile) heart rates of 84 (59–110) and 75 (55–101) beats per minute at day 1 and 14 post-partum, respectively.^6^

There is a paucity of literature on maternal bradycardia. Published information is currently limited to case reports, with the underlying etiologies attributed primarily to intraoperative vasoactive medications and neuraxial anesthesia. However, these factors are less likely to be contributors when maternal bradycardia occurs post-partum. The objective of this study was to describe the characteristics, underlying associated conditions, and clinical course of women who develop bradycardia post-partum from our institution. As a secondary objective, we conducted a literature review to summarize all available cases of post-partum bradycardia reported to date.

## Materials and methods

### Chart review

British Columbia Women's Hospital (BCWH) is the largest stand-alone maternity care hospital in Vancouver, Canada, with approximately 7400 deliveries annually. The Obstetrical Medicine service provides inpatient and outpatient consultations to women with medical conditions during pregnancy, including those with low risk cardiac disease. Hypertensive disorders of pregnancy are the most common reason for referral by the Obstetrical Medicine consult service (> 500 consults per year). Retrospective chart review was completed in duplicate by two study investigators (KT, CF) of all women who were referred to the BCWH Obstetrical Medicine service from January 2012 to May 2020, inclusive. Case ascertainment was identified from our Obstetrical Medicine database. We included all referrals of maternal bradycardia cases with heart rate less than 60 beats per minute sustained for greater than 10 min or those who required intervention at any time in the post-partum period up to 30 days. Patients were excluded if bradycardia started more than 1-month post-partum or had bradycardia prior to pregnancy.

We extracted baseline demographic data including: age, parity, gestational age at delivery, past comorbidities, anesthetic administered at time of delivery, mode of delivery, and medications. We collected baseline heart rate, blood pressure (BP), blood work, and cardiac diagnostics including electrocardiogram (ECG), echocardiogram, and ambulatory ECG monitor results. The duration of bradycardia was determined by calculating the start and end time of bradycardia based on vital sign records, and rounded to the nearest day, as continuous cardiac monitoring was not routinely performed in our institution. We designated a possible associated condition of the bradycardia (i.e. medications, neuraxial anesthesia, preeclampsia, underlying, cardiac, or other) based on assessment of comorbid conditions, clinical course in hospital, investigations and follow up through independent review by two physicians (KT, CF). We classified the potential association of bradycardia with medications or neuraxial anesthesia related if participants were taking a known medication that can cause bradycardia or if bradycardia occurred shortly after administration of medication or anesthesia. Bradycardia that was postulated to be associated with preeclampsia was in women with BP greater than 140/90 mm Hg and urine protein to creatinine ratio greater than 30 mg/mmoL as per guidelines.^7^ Bradycardia associated with preeclampsia also included normal cardiac investigations and timing of bradycardia to be unrelated to medications or neuraxial anesthesia. Underlying bradycardia was defined as bradycardia that did not resolve at 6 weeks follow up with normal cardiac investigations and normal BP. An underlying cardiac cause of the bradycardia was postulated if participants had abnormal echocardiography or abnormalities on ambulatory ECG monitoring. If a possible association could not be determined, it was classified as other. Any discrepancies were resolved by discussion and assessment by a third physician adjudicator (WSC).

### Description of search concepts

We completed a literature review by performing a search of the English language using the key words “bradycardia,” “pregnancy,” “arrhythmia,” “post-partum,” and “maternal” in MEDLINE Ovid (inception to May 2020), and Embase Ovid (1974 to June 2022). We limited our search to case reports, case series, and conference proceedings. We did not review case reports where pregnant women had known underlying congenital heart arrhythmias or heart block.

### Statistical analysis

Descriptive statistics was used to analyze baseline data. Data was expressed as mean  ±  standard deviation and median (interquartile range (IQR)).

## Results

In total, we identified 24 women who were referred to the Obstetrical Medicine service for post-partum bradycardia. The mean age was 34.2  ±  4.8 years. Approximately 29% of the patients were nulliparous. The proportion of patients that delivered vaginally and by caesarean section were 45.8%, and 54.2%, respectively. Spinal and epidural anesthesia was administered to 20.8% and 70.8% of the participants, respectively. The proportion of pregnancies complicated by hypertensive disorders of pregnancy and gestational diabetes were 45.8% and 16.7%, respectively. Baseline demographic information is summarized in Table 1. Detailed description of post-partum maternal bradycardia cases is summarized in Table 2.

**Table 1.** Baseline demographics of women with post-partum bradycardia and symptoms experienced.

Baseline demographics *N* (%)	
Age (Mean)	34.2 ± 4.8 years
Nulliparous	7 (29.1%)
Gestational age at delivery	271 ± 12.8 days
Spinal/epidural anesthetic	22 (91.7%)
Mean heart rate (beats per minute)	40.4 ± 8.1
Systolic blood pressure (mmHg)	131 ± 24
Diastolic blood pressure (mmHg)	72 ± 11
Symptomatic	17 (70.8%)
Time to resolution^a^	3.6 ± 3.8 days
**Symptoms experienced**	
Shortness of breath	5 (29.4%)
Chest pain	4 (23.5%)
Dizziness	3 (17.6%)
Headaches	3 (17.6%)
Edema	3 (17.6%)
Fatigue	2 (11.8%)

^a^ Data unavailable for 5 patients (20.8%).

**Table 2.** Cases of post-partum bradycardia based on presumed etiology

**Demographics**	**Heart rate and rhythm**	**Duration of bradycardia**	**BP at lowest heart rate**	**Symptoms**	**Comments**
**Preeclampsia**					
36F G4P1, forceps delivery at 40 weeks	45 bpm, sinus bradycardia	3 days	136/80	Dyspnea	
37F G1P1, C-section delivery at 39 weeks	50 bpm, sinus bradycardia	4 days	176/84	Headache	
32F G2P1, SVD at 36+2 weeks	40 bpm, sinus bradycardia	1 day	110/66	Edema	Twin pregnancy
36F G4P4, SVD at 39+1 weeks	45 bpm, sinus bradycardia	1 day	145/86	Headache	
36F G2P2, C-section at 41+3 weeks	40 bpm, sinus bradycardia	Unknown	134/73	Fatigue	
29F G7P5, SVD at 39 weeks	38 bpm, sinus bradycardia	Unknown	144/84	None	Echocardiogram showed tricuspid valve infective endocarditis
26F G1P1, forceps delivery at 39+1 weeks	44 bpm, sinus bradycardia	7 days	135/75	Chest pain	
32F G2P1, SVD at 36+2 weeks GA, twin pregnancy	40 bpm, sinus bradycardia	1 day	100/54	None	
39F G3P2, C-section at 40+3 weeks	38 bpm, sinus bradycardia	6 days	176/88	Scotoma, Dyspnea pedal edema	
34F G3P3, C-section at 36 weeks	38 bpm, first degree AV block	11 days	149/70	Dizziness	
39F G2P2, C-section, at 39 weeks	50 bpm, sinus bradycardia	1 day	155/77	Chest pain, Dyspnea	
37F G2P1, C-section at 40 weeks	52 bpm, sinus bradycardia	10 days	183/95	Dyspnea, pedal edema	
39F G2P1, C-section at 38+1 weeks	45 bpm, sinus bradycardia	3 days	145/84	Chest pain	
**Pre-existing**					
42F, G3P2, C-section at 39+4 weeks	20 bpm, sinus bradycardia	Not resolved at follow up	100/60	None	
28F, G1, C-section at 38+3 weeks	40 bpm, sinus bradycardia	Not resolved at follow up	125/60	None	
38F, G4P2, SVD at 39+4 weeks	37 bpm, trigeminy	1 day	109/53	None	
34F, G4P1, C-section at 38 weeks	33 bpm, sinus bradycardia	Not resolved at follow up	116/75	None	
**Medications**					
28F G2P1, vacuum delivery at 39+5 weeks	40 bpm, sinus bradycardia	1 day	113/81	Lightheaded	Ergotamine in OR
34F G2P2, SVD at 39 weeks	20 bpm, bigeminy/trigeminy	1 day	108/67	Fatigue	Ergotamine in OR
**Anesthetic**					
22F G1, forceps delivery at 38 weeks	50 bpm, Mobitz Type I heart block	Immediate	110/60	Lightheaded	Spinal anesthetic
40F, G2, C-section delivery at 38+1 weeks	45 bpm, sinus bradycardia	1 day	108/70	Fatigue	Spinal anesthetic
**Cardiac**					
33F G1, SVD at 37+6 weeks GA	40 bpm, bigeminy	2 days	110/58	Lightheaded	Echocardiogram: EF 45%
**No etiology identified**					
35F G1, C-section at 41+3 weeks GA	33 bpm, sinus bradycardia	7 days	136/67	None	Received antibiotics and repeat operation for infected C-section wound
35F G2P1, SVD at 39+6 weeks	40 bpm, sinus bradycardia	5 days	124/70	Dyspnea	Chinese herbal medicine

Abbreviation: GA = gestational age; bpm = beats per minute; BP=blood pressure; SVD = spontaneous vaginal delivery; C-section = caesarean section; OR = operating room; EF = ejection fraction

The mean baseline heart rate prior to delivery was 76.1  ±  12.3 beats per minute. At the time of bradycardia presentation, the mean heart rate was 40.1  ±  6.4 beats per minute; the mean systolic and diastolic BP was 131  ±  24 and 72  ±  11 mm Hg, respectively. Approximately 70% of participants were symptomatic, with the most common symptoms being dyspnea (29.4%), chest pain (23.5%), dizziness (17.6%), headaches (17.6%), and edema (17.6%). There were no participants that had syncope as their primary symptom of bradycardia. Bradycardias were commonly associated with preeclampsia (54.1%), underlying conditions (16.7%), medications (8.3%), neuraxial anesthesia (8.3%), and underlying cardiac conduction or structural heart disease (4.2%) (Table 3). Two cases had no associated condition identified. Common medication implicated in bradycardia included Chinese herbal products and ergotamine.

**Table 3.** Cardiac rhythm, and associated conditions, and diagnostic investigations in women with post-partum bradycardia.

Cardiac rhythm	
Sinus bradycardia	19 (79.2%)
Bigeminy/trigeminy	3 (12.5%)
First degree heart block	1 (4.2%)
Mobitz Type 1 block	1 (4.2%)
**Associated conditions**	
Preeclampsia	13 (54.1%)
Underlying	4 (16.7%)
Medications	2 (8.5%)
Anesthesia	2 (8.3%)
Cardiomyopathy	1 (4.2%)
None identified	2 (4.2%)

The median time to presentation of bradycardia in the post-partum period was 3 days, but ranged from immediately post-partum to 22 days. We observed that bradycardia associated with neuraxial anesthesia occurred almost immediately post-partum, either in the operating room or post-anesthesia recovery room, whereas bradycardia associated with preeclampsia occurred a median of 4 (IQR 3–8) days post-partum.

The most common rhythm identified on ECG was sinus bradycardia (79.2%), followed by bigeminy/trigeminy (12.5%), first-degree heart block (4.2%), and Mobitz type I (4.2%) (Table 3). Given that continuous cardiac monitoring was not performed, with the data available, mean and median time to resolution of bradycardia was 3.6  ±  3.8 days and 1 (IQR 1–3) days, respectively. In cases presumed secondary to anesthesia, resolution of bradycardia ranged from hours to 1 day. In contrast, cases presumed secondary to preeclampsia the median time to resolution of bradycardia was 3 (IQR 1–7) days. Post-partum hypertension normalized with resolution of bradycardia.

Three women (12.5%) received magnesium sulfate during their hospitalization, of which two cases were indicated for severe preeclampsia and one case was indicated for hypomagnesemia. No women received labetalol for hypertension management. The most common anti-hypertensive medication prescribed was oral extended-release nifedipine. One woman received atropine and one received glycopyrrolate for treatment of severe bradycardia with minimum heart rates of 20 and 20–36 beats per min, respectively. None of the women required pacemaker insertion or transfer to a higher level of care.

The most common investigations completed for work up of bradycardia included troponin, brain natriuretic peptide (BNP), and TSH, which were completed 75%, 58.3%, and 54.2%, of the time (Table 4). Only one woman had an abnormal troponin level (0.44 µg/L, normal <0.05 µg/L). Her echocardiogram and remaining cardiac work up were normal. The mean BNP was 153.3  ±  88.6 ng/L and TSH was 2.43  ±  1.65 mU/L. A total of 15 women (62.5%) had echocardiograms, of which 13 were reported as normal. One woman had a finding of tricuspid valve vegetation, suggestive of infective endocarditis. This was felt to be an incidental finding as the patient's bradycardia improved spontaneously and she was afebrile. One woman had an echocardiogram report that demonstrated a dilated cardiomyopathy with an ejection fraction of 45%, in association with a baseline ECG rhythm of bigeminy. Ambulatory ECG monitoring was performed in 29.2% of the participants after discharge, all demonstrating resolution of bradycardia.

**Table 4.** Summary of results of cardiac investigations.

Diagnostic tests and investigations performed	
Transthoracic echocardiogram	15 (62.5%)
Ambulatory ECG monitor	7 (29.2%)
BNP	13 (54.2%)
Troponin	18 (75%)
TSH	14 (58.3%)
**Echocardiogram, *n* (%)**	
Not completed	9 (37.5%)
Normal	13 (54.2%)
Dilated cardiomyopathy	1 (4.2%)
Tricuspid valve vegetation	1 (4.2%)
**Cardiac biomarkers and thyroid testing**	
Average BNP (pg/mL)	153.3 ± 88.6
Proportion of troponin abnormal	1 (4.2%)
Average TSH (mU/L)	2.43 ± 1.65

BNP: brain natriuretic peptide; TSH: thyroid stimulating hormone.

Our literature review revealed 14 case reports of pregnant women with bradycardia (*N*  =  28 pregnant women). The majority of case reports accounted for one pregnant woman. One case series, reported in grey literature (non-published), included 15 pregnant women. Case reports are summarized in Table 5. Heart rates ranged from 29 to 51 beats per minute. Bradycardia resolved after 1.5 to 24 h, depending on the associated condition. It was noted that if bradycardia was presumed secondary to anesthesia it would resolve within hours. The most common etiologies described by the authors for maternal bradycardia was medications,^8–12^ primary conduction abnormalities,^5,13,14^ preeclampsia,^15,16^ anesthesia,^17^ and peripartum cardiomyopathy.^18^ Two case reports did not identify a clear cause for the patient's bradycardia.^19,20^

**Table 5.** Summary of post-partum maternal bradycardia literature.

Author	Demographics	Heart rate/rhythm	Duration of bradycardia	Mode of delivery	Etiology	Comments
**Medications**						
Abuladze et al. 2018 (8)	30F G1, 40+3 weeks, spontaneous rupture of membranes	29-42 bpm ECG: sinus rhythm with PAC and PVC	3 hours after discontinuing oxytocin	C-section	Oxytocin	Asymptomatic
Sarumi et al. 2019 (10)	32F G2P1, 36+4, intrauterine growth restriction	41-59bpm, ECG: sinus bradycardia	49 hours	C-section	Betamethasone	Asymptomatic, occurred antepartum
Ibrahim et al. 2008 (11)	30F G6P5, 37+2 weeks, twin pregnancy,	36bpm, ECG: sinus bradycardia	90 min	SVD	Methylergonovine maleate	Symptoms: presyncope, nausea, vomiting, chest pain.
Hennessy et al. 1999 (9)	37F G1, 33 weeks with preeclampsia	44 (39-45) bpm, ECG: sinus bradycardia with T wave inversions, U waves	Post-partum day 2	C-section	Magnesium sulfate (1.58 mmol/L) and preeclampsia	Symptoms: Headache Echocardiogram normal
Garg et al. 2015 (12)	28F G1P1, PPD 8 with eclampsia	51 bpm, ECG: sinus bradycardia	N/A	SVD	Eclampsia, Magnesium sulfate	Symptoms: Headaches, seizure
**Anesthesia**						
Pan et al. 2004 (17)	27F G2P0, 34 weeks	30 bpm, ECG asystole	Rapid after interventions of CPR, laterally tilted position, atropine 1 mg	SVD	Combined spinal epidural anesthetic	Bradycardia and asystole occurred 35 min after intrathecal injection of sufentanil
**Cardiac/Conduction abnormality**						
Nof et al. 2011 (13)	15 women	ECG: 42 ± 5 bpm; Holter: 54 ± 9 bpm	Rapid recover post-partum (duration unclear)	N/A	3/15: missense mutation in *HCN4* 2/15: *M1113V* mutation 1/15: *A485V* mutation	Symptoms: mild Echocardiogram normal
Codsi et al. 2017 (18)	28F G1P1, PPD5	30-40 bpm, ECG: sinus bradycardia	Intermittent sinus bradycardia 40 bpm up to 2 month post-partum	SVD	Peripartum cardiomyopathy, query secondary to high parasympathetic tone	BP initially 158/77, preeclampsia work up negative, Echocardiogram 35% with regional wall motion abnormality, cardiac catheterization 20% LAD lesion, no SCAD
Adekanye et al. 2007 (5)	28F G1, chronic hypertension at 38 weeks	38 (baseline 45-55) bpm, ECG: complete heart block, escape rhythm	Received pacemaker at 6 weeks post-partum	SVD	Congenital complete heart block	Echocardiogram normal
Joseph et al. 2010 (14)	28F G1, 36 weeks	40-50 bpm ECG: complete heart block	Pacemaker inserted	C-section	Complete heart block not yet diagnosed, query edema in myocardium	Heart rate before delivery 76 bpm
**Preeclampsia**						
Korzets et al. 1994 (15)	28F G3P2, PPD5	35 bpm, ECG: sinus bradycardia	Recovered after magnesium sulfate, recurred when BP > 180/110 post-PPD5	SVD	Eclampsia secondary to increased intracranial pressure	Symptoms: Headache, seizure CT head normal
Angsubhakorn et al. 2021 (16)	34F G3P3, PPD3	64 bpm, ECG: sinus rhythm	Recovered after intravenous furosemide and normalization of BP	SVD	Acute pulmonary edema from preeclampsia	Symptoms: shortness of breath Echocardiogram normal
**No Identifiable Etiology**						
Devendra et al. 2006 (19)	32F G1, healthy	40 bpm, ECG: sinus bradycardia	3 hours after delivery	SVD	Unclear etiology, occurred during labour	Symptoms: Dizziness Echocardiogram normal
Hosokawa et al. 2017 (20)	36F G1, 28+6 weeks, preeclampsia	48 bpm, ECG: sinus bradycardia	2 hours	C-section	Unknown, query dysregulation of sympathetic and parasympathetic nervous system, HELLP	Tachycardia (160 bpm) and BP 201/111 after bradycardia resolved (intrapartum)

Abbreviation: BP = Blood pressure; C-section = caesarean section; bpm = beats per minute; PAC = premature atrial contraction; PVC = premature ventricular contraction; SVD = spontaneous vaginal delivery; PPD = post-partum day; LAD = left anterior descending artery; SCAD = spontaneous coronary artery dissection

## Discussion

The key findings of our analysis of maternal post-partum bradycardias: (1) they are benign, self-limited, and rarely require intervention, (2) their onset and duration can be variable depending on the associated condition, and (3) preeclampsia is a common associated condition.

We observed that the majority of bradyarrhythmias in the post-partum period are self-limited and benign (sinus bradycardia). Most women were symptomatic, but the majority of bradycardias resolved without any treatment interventions. The majority of women had normal investigations including echocardiogram, ambulatory ECG monitoring, and cardiac biomarkers. From our literature review, two cases were noted to require a permanent pacemaker insertion, which led to a new diagnosis of underlying congenital heart block.^5,21^ All pregnant women with bradycardia require an ECG to assess the underlying cardiac rhythm.

Interestingly, the onset and duration of post-partum bradycardia differed between associated conditions. For instance, post-partum neuraxial anesthetics and medications occurred almost immediately after receiving the pharmacological agent. We observed that the duration of bradycardias secondary to anesthesia were short (hours to 1 day). In contrast, preeclampsia associated bradycardia occurred a median of 4 (IQR 3–8) days post-partum, and could persist up to 10 days. This delayed onset of post-partum bradycardia mirrors the fact that BP typically increases post-partum days 3 to 5.^22^ It is important for clinicians to recognize that bradycardia can persist for several days, but often will resolve without clinical interventions.

We observed that bradycardia secondary to anesthesia occurred in 8.3% of our cases, which is similar to the literature, which reports that regional or general anesthesia at time of caesarean section is associated with maternal bradycardia in 10–13% of cases.^23,24^ From our case series, ergotamine was the most common medication suspected of causing bradycardia. Pharmacological associations of maternal bradycardia described in the literature include magnesium sulfate,^9,12^ betamethasone,^10^ ergotamine,^11^ and anesthetics.^25^ We observed that when bradycardia was associated with medications or anesthesia, the time to recovery was much shorter than when it is associated with preeclampsia, underlying or cardiac causes.

In our case series, preeclampsia was identified as an associated condition for women's bradycardia, followed by underlying, medications and anesthesia. In two cases, no clear associated condition of bradycardia could be identified. This differs from what has been previously reported in the literature, where more cases of post-partum bradycardia were presumed secondary to medications^8–12^ and primary cardiac or conduction abnormalities.^5,13,14^ Only two cases attributed preeclampsia as the primary associated condition of bradycardia.^15,16^ Interestingly, of all the case reports identified (15 cases), 5 women had complications of hypertensive disorders of pregnancy, including preeclampsia,^9^ eclampsia^12,15^ and hemolysis, elevated liver enzymes, low platelets syndrome.^20^ Two case reports identified magnesium sulfate as a possible reason for bradycardia^12,15^; however, maternal bradycardia is not a well-recognized side effect of magnesium sulfate for obstetric indications, unless at supra-therapeutic levels.^26^ Therefore, we postulate that preeclampsia may have predisposed the women to bradycardia, which was observed in our case series.

The pathophysiology for how preeclampsia is associated with bradycardia is unknown. Potential hypotheses include Cushing response, characterized by hypertension and bradycardia associated with increased intracranial pressure, as a compensatory mechanism to minimize ischemia in the brain through activation of the baroreceptors of carotid bodies.^27^ In our literature review, two women were diagnosed with eclampsia, and therefore, increased intracranial pressure may be responsible for bradycardia due to the Cushing response in these cases. It is difficult to prove this mechanism, as the majority of women with preeclampsia were not investigated for increased intracranial pressure. Another potential mechanism is posterior reversible encephalopathy syndrome, which is commonly associated with preeclampsia and eclampsia due to loss of cerebral autoregulation.^24^ This was not investigated as a potential associated condition in our series or in the literature we identified, and may be under-reported due to under-diagnosis. Other potential mechanisms hypothesized include the effect of preeclampsia mediating increased vagal tone via arterial and cardiac baroreceptors.^16^ Furthermore, as preeclampsia is commonly associated with edema, it is hypothesized that edema may affect the cardiac conduction system to cause bradyarrhythmias, however, studies have not demonstrated this.^14^

Data is conflicting on pulse adaptations in preeclampsia. Compared with normotensive women, preeclampsia is associated with significantly lower heart rates in the third trimester (85  ±  10 versus 71  ±  14 beats per minute).^28^ The exact mechanism for this is unclear. Other studies have shown that preeclampsia is associated with higher heart rates than normotensive pregnant females (90.88  ±  14.57 vs 69.02  ±  12.72 beats per minute).^29^ Previous studies have postulated that preeclampsia is related to dysregulation of the autonomic nervous system with exaggerated sympathetic activity and attenuated parasympathetic activity.^29^ In the post-partum period, bradycardia is uncommon, where median (3rd–97th centile) heart rates are 84 (59–110) and 75 (55–101) beats per minute at day 1 and 14 post-partum, respectively, in a large prospective cohort of post-partum women.^6^

Our study is the largest case series to date on maternal bradycardia in the post-partum period. Our case series represents those from a single center, that represent women with low risk cardiac disease, which makes our results more generalizable compared to other case studies that involve women with complex structural heart disease. We were able to provide follow-up data on the majority of patients to ensure that symptoms and bradycardia resolved in the post-partum period. The primary limitation of our study is referral bias, as the majority of the patients referred to the Obstetrical Medicine services are women with preeclampsia and other hypertensive disorders of pregnancy. It is possible that women with more benign etiologies of maternal bradycardia were not referred as their bradycardias were transient and women were asymptomatic. Acute and transient bradycardias may be more likely to be managed by anesthesiologists; however, once patients are assessed and stabilized, these patients are usually referred to Obstetrical Medicine service in our institution to workup the underlying cause, especially if there is persistence of bradycardia in the post-partum period. Secondly, we did not conduct a review of the grey literature, and we may not have captured all cases that have been described. Finally, the majority of women did not have continuous cardiac monitoring, and therefore the duration of bradycardia may be overestimated.

## Conclusions

Maternal bradycardia is an uncommon condition complicating pregnancy. Maternal bradycardia rarely persists beyond 7 days post-partum, supporting conservative management. The majority of maternal bradycardia is associated with preeclampsia. It is important for clinicians caring for pregnant women to be aware of the association with maternal bradycardia and preeclampsia.
